# Degree sequence for *k*-arc strongly connected multiple digraphs

**DOI:** 10.1186/s13660-017-1544-3

**Published:** 2017-10-25

**Authors:** Yanmei Hong, Qinghai Liu

**Affiliations:** 1 College of Mathematics and Computer Science, Fuzhou University, Fuzhou, 350108 P.R. China; 2 Center for Discrete Mathematics, Fuzhou University, Fuzhou, 350108 P.R. China

**Keywords:** degree sequence, realization, *k*-arc strongly connected

## Abstract

Let *D* be a digraph on $\{v_{1},\ldots, v_{n}\}$. Then the sequence $\{ (d^{+}(v_{1}), d^{-}(v_{1})), \ldots, (d^{+}(v_{n}), d^{-}(v_{n}))\}$ is called the degree sequence of *D*. For any given sequence of pairs of integers $\mathbf{d}=\{(d_{1}^{+}, d_{1}^{-}), \ldots, (d_{n}^{+}, d_{n}^{-})\}$, if there exists a *k*-arc strongly connected digraph *D* such that **d** is the degree sequence of *D*, then **d** is realizable and *D* is a realization of **d**. In this paper, characterizations for *k*-arc-connected realizable sequences and realizable sequences with arc-connectivity exactly *k* are given.

## Introduction

Digraphs in this paper may have loops and parallel arcs. A digraph *D* is called a *multiple digraph* (or *multi-digraph* for short) if it has no loops. Furthermore, if *D* has parallel arcs neither, then *D* is *strict*. We follow [1] for undefined terminologies and notation.

For a digraph *D*, as in [1], $V(D)$ and $A(D)$ denote the vertex set and the arc set of *D*, respectively; and $(u,v)$ represents an arc oriented from a vertex *u* to a vertex *v*. For any two disjoint vertex sets *X* and *Y*, let $A(X,Y) =\{(u,v) \in A(D)| x \in X, y \in Y\}$. For a subset $X \subseteq V(D)$, define

$$\partial_{D}^{+}(X) = A \bigl(F, V(D) \setminus X \bigr)\quad \mbox{and}\quad \partial_{D}^{-}(X) = \partial_{D}^{+} \bigl(V(D)\setminus X \bigr). $$

We use $D[X]$ to denote the subdigraph of *D* induced by *X*. If *F* is a subdigraph of *D*, then for notational convenience, we often use $\partial_{D}^{+}(F), \partial_{D}^{-}(F)$ for $\partial_{D}^{+}(V(F)), \partial _{D}^{-}(V(F))$, respectively.

For a vertex *u* of *D*, define the *out-degree* $d_{D}^{+}(u)$ (*in-degree* $d_{D}^{-}(u)$, respectively) of *u* to be $\vert \partial_{D}^{+}(\{u\}) \vert $ ($\vert \partial_{D}^{-}(\{u\}) \vert $, respectively). Let $V(D)=\{v_{1},\ldots,v_{n}\}$. The sequence of integer pairs $\{(d_{D}^{+}(v_{1}),d_{D}^{-}(v_{1})), (d_{D}^{+}(v_{2}),d_{D}^{-}(v_{2})), \ldots ,(d_{D}^{+}(v_{n}),d_{D}^{-}(v_{n}))\}$ is called a *degree sequence* of *D*. For a given sequence $\mathbf {d}=\{(d_{1}^{+},d_{1}^{-}),\ldots,(d_{n}^{+},d_{n}^{-})\} $, to determine whether there is a digraph *D* such that *D* has degree sequence **d** is a very essential problem in graph theory. This problem is closely linked with the other branches of combinatorial analysis such as threshold logic, integer matrices, enumeration theory, etc. The problem also has a wide range of applications in communication networks, structural reliability, stereochemistry, etc.

For a digraph *D*, if for any ordered pair of vertices $(u,v)$, there is a directed path from *u* to *v*, then *D* is said to be *strongly connected*. Characterizations for a digraphic sequence and a multi-digraphic sequence with realizations having prescribed strong arc-connectivity have been studied, see Frank [2, 3] and Hong *et al.* [4]. For more in the literature on degree sequences, see surveys [5] and [6].

A sequence of integer pairs $\mathbf {d}=\{(d_{1}^{+},d_{1}^{-}),\ldots,(d_{n}^{+},d_{n}^{-})\}$ is *digraphic* (*multi-digraphic*, respectively) if there exists a digraph (a multi-digraph, respectively) *D* with degree sequence **d**, where *D* is called a **d**-realization. Let $\langle\mathbf{d}\rangle$ be the set of all **d**-realizations. Frank [2, 3] (see also Theorem 63.3 in [7]) showed that $\langle\mathbf {d}\rangle\neq\emptyset$ if and only if $\sum_{i=1}^{n} d_{i}^{+} =\sum_{i=1}^{n} d_{i}^{-}$. If a multi-digraphic realization of **d** is required, then Hong *et al.* [4] gave the following characterization.

### Theorem 1.1

(Hong, Liu, Lai)

*Let* $\mathbf {d}=\{(d_{1}^{+},d_{1}^{-}),\ldots,(d_{n}^{+},d_{n}^{-})\}$ *be a sequence of non*-*negative integer pairs*. *Then* **d** *is multi*-*digraphic if and only if each of the following holds*:

(i) $\sum_{i=1}^{n} d_{i}^{+} =\sum_{i=1}^{n} d_{i}^{-}$;
(ii) $\textit{for }k=1,\ldots, n,d_{k}^{+} \leq\sum_{i\neq k} d_{i}^{-}$.

Furthermore, for a strict digraph, there is a similar result. The following theorem, which can be found in [8–10] among others, is well known.

### Theorem 1.2

(Fulkerson-Ryser)

*Let* $\mathbf {d}=\{(d_{1}^{+},d_{1}^{-}),\ldots,(d_{n}^{+},d_{n}^{-})\}$ *be a sequence of non*-*negative integer pairs with* $d_{1}^{+}\geq\cdots\geq d_{n}^{+}$. *Then* **d** *is strict digraphic if and only if each of the following holds*:

(i) $d_{i}^{+}\leq n-1, d_{i}^{-}\leq n-1\textit{ for all }1\leq i\leq n$;
(ii) $\sum_{i=1}^{n} d_{i}^{+} =\sum_{i=1}^{n} d_{i}^{-}$;
(iii) $\sum_{i=1}^{k} d_{i}^{+} \leq\sum_{i=1}^{k} \min\{k-1,d_{i}^{-}\} + \sum_{i=k+1}^{n} \min\{k,d_{i}^{-}\}\textit{ for all }1\leq k\leq n$.

Let *D* be a digraph and *k* be an integer. If for any arc set *S* of *D* with $\vert S \vert < k$, $G-S$ is still strongly connected, then *D* is said to be *k-arc strongly connected* (or *k-arc-connected* for short). Clearly, 1-arc connected digraph is also a strongly connected digraph and vice versa. The *arc-connectivity* of *D*, denoted by $\lambda(D)$, is the maximum integer *k* such that *D* is *k*-arc-connected. In [4], Hong *et al.* characterized the sequence of pairs of integers **d** so that there is a strongly connected digraph $D\in\langle\mathbf{d}\rangle$. Also, they gave an example to point out that to characterize the case whether there is a *k*-arc-connected digraph in $\langle\mathbf {d}\rangle$ may be very difficult. In this paper, we consider a multi-digraphic version. We will give a characterization for *k*-arc-connected multi-digraphs. Furthermore, we also give a characterization for multi-digraphs with arc-connectivity exactly *k*.

In the next section, we will give some tools and methods used in this paper. In Section 3, we characterize the sequence of pairs of integers to have a *k*-arc-connected realization. In Section 4, we characterize the sequence of pairs of integers to have a realization that has arc-connectivity exactly *k*. In Section 5, we give a conclusion of this paper.

## Methods and tools

In this section, we give a special notation used in this paper that is also the main tool. Let *D* be a digraph and $(u_{1},v_{1}), (u_{2}, v_{2})$ be two arcs of *D*. The *2-switch* of *D* is an operation to obtain a new digraph $D'$ from $D - \{(u_{1},v_{1}), (u_{2}, v_{2})\}$ by adding $\{(u_{1},v_{2}), (u_{2},v_{1})\}$. The resulting digraph $D'$ is often denoted by $D \otimes\{(u_{1},v_{1}), (u_{2},v_{2})\}$. By this definition,

1 $$ \mbox{$D\otimes \bigl\{ (u_{1},v_{1}), (u_{2},v_{2}) \bigr\} $ and $D$ have the same degree sequence.} $$

Thus, the degree sequence remains unchanged under 2-switch operations. This operation will be the main tool in the arguments of this paper.

Note that in the operation of 2-switch, the two arcs $(u_{1},v_{1})$, $(u_{2},v_{2})$ may have common ends. For example, if $u_{1}=u_{2}$ or $v_{1}=v_{2}$, then the resulting digraph is exactly the same as the original digraph. If $v_{1}=u_{2}$ or $v_{2}=u_{1}$, then the resulting digraph has loops. So, when this case occurs, we usually use another 2-switch operation to remove the loops. For example, assume $(x,y), (y,z),(u,v)\in A(D)$ and $(D\otimes\{(x,y),(y,z)\})\otimes\{(y,y), (u,v)\}$ is just the digraph $D-\{(x,y), (y,z), (u,v)\}+\{(x,y),(u,y),(y,v)\}$. After these two 2-switches, the resulting digraph still lies in $\langle\mathbf {d}\rangle$. In this paper, we will use these operations to obtain a *k*-arc-connected digraph or a digraph with arc-connectivity exactly *k* from an arbitrary digraph in $\langle\mathbf {d}\rangle$.

Let $\mathbf {d}=\{(d_{1}^{+}, d_{1}^{-}),\ldots, (d_{n}^{+}, d_{n}^{-})\}$. By using the tools and the methods above, we obtain a sufficient and necessary condition of **d** to have a *k*-arc-connected realization (see Theorem 3.1). Furthermore, if we require the realization *D* to have arc-connected exactly *k*, then we get Theorem 4.1.

## Degree sequence for *k*-arc-connected multi-digraphs

In this section, we shall present a characterization for multi-digraphic sequences with *k*-arc-connected realizations. We will give some notations used in this section fist.

Let *D* be a digraph. For a subset $F \subseteq V(D)$, define $\overline{F} = V(D) \setminus F$. A vertex set $F \subseteq V(D)$ is called an *out-fragment* (*in-fragment*, respectively) of *D* if $\vert \partial_{D}^{+}(F) \vert = \lambda(D)$ ($\vert \partial_{D}^{-}(F) \vert = \lambda(D)$, respectively). Both out-fragments and in-fragments are also called fragments of *D*. An out-fragment (in-fragment, respectively) *F* is *minimal* if any proper subset of *F* is no longer an out-fragment (in-fragment, respectively). Let $\mathrm{fr}^{+}(D)$ be the number of out-fragments of *D* and $\mathrm{fr}^{-}(D)$ be the number of in-fragments of *D*. As a vertex set *F* is an out-fragment if and only if its complement *F̅* is an in-fragment, $\mathrm{fr}^{+}(D)=\mathrm{fr}^{-}(D)$. Denote $\mathrm{fr}(D)=\mathrm{fr}^{+}(D)=\mathrm{fr}^{-}(D)$. It is easy to see that $\mathrm{fr}(D)>0$ for any digraph *D*. This observation can be used to prove the following theorem.

### Theorem 3.1

*Let* $\mathbf {d}=\{(d_{1}^{+},d_{1}^{-}),\ldots,(d_{n}^{+},d_{n}^{-})\}$ *be a sequence of integer pairs*. *Then* **d** *has a* *k*-*arc*-*connected realization if and only if each of the following holds*:

(i) $\sum_{i=1}^{n} d_{i}^{+}=\sum_{i=1}^{n} d_{i}^{-}$;
(ii) *for each* $1\leq j\leq n$, $d_{j}^{+}, d_{j}^{-}\geq k$;
(iii) *for each* $1\leq j\leq n$, $d_{j}^{+}\leq\sum_{i\neq j}d_{j}^{-}$.

### Proof

If **d** has a *k*-arc-connected realization, then by Theorem 1.1, (i) and (iii) hold, and by the definition of *k*-arc-connectedness, (ii) holds. So, it suffices to prove the sufficiency. By (i), (iii) and by Theorem 1.1, $\langle\mathbf {d}\rangle\neq\emptyset$. So we may pick a multi-digraph $D\in\langle\mathbf {d}\rangle$ such that

2 $$ \textstyle\begin{array}{ll} \mbox{(a) the arc-connectivity $\lambda(D)$ is as large as possible.}\\ \mbox{(b) subject to (a), $\mathrm{fr}(D)$ is as small as possible.} \end{array} $$

We shall show that *D* is *k*-arc-connected. Suppose this is not true. Then $\lambda(D)< k$.

By the definition, $\mathrm{fr}(D)>0$. Then there exist out-fragments and in-fragments in *D*. Let $F_{1}$ be a minimal out-fragment of *D* and $F_{2}$ be a minimal in-fragment contained in $\overline{F_{1}}$. Then $\vert \partial_{D}^{+}(F_{1}) \vert = \vert \partial_{D}^{-}(F_{2}) \vert =\lambda(D)$. By (ii), $d_{i}^{+},d_{i}^{-}\geq k>\lambda(D)$, and so there must be $u_{1}, v_{1} \in F_{1}$ and $u_{2}, v_{2} \in F_{2}$ such that $(u_{1}, v_{1}) \in A(D[F_{1}])$ and $(u_{2},v_{2}) \in A(D[F_{2}])$. Let $D'=D\otimes\{(u_{1},v_{1}),(u_{2},v_{2})\}$. By (1), $D'$ is also a multi-digraph in $\langle\mathbf {d}\rangle$.

*Claim* 1. If *F* is an out-fragment of $D'$, then one of the following must hold:

(i) $\vert \partial_{D'}^{+}(F) \vert = \vert \partial_{D}^{+}(F) \vert $, or
(ii) $\partial_{D'}^{+}(F) = \partial_{D}^{+}(F) \cup\{(u_{2}, v_{1})\}$ and $F \cap\{u_{1}, v_{1}, u_{2}, v_{2}\} = \{u_{2}, v_{2}\}$, or
(iii) $\partial_{D'}^{+}(F) = \partial_{D}^{+}(F) \cup\{(u_{1}, v_{2})\}$ and $F \cap\{u_{1}, v_{1}, u_{2}, v_{2}\} = \{u_{1}, v_{1}\}$, or
(iv) $\partial_{D'}^{+}(F) = \partial_{D}^{+}(F) - \{(u_{2}, v_{2})\}$ and $F \cap\{u_{1}, v_{1}, u_{2}, v_{2}\} = \{u_{2}, v_{1}\}$, or
(v) $\partial_{D'}^{+}(F) = \partial_{D}^{+}(F) - \{(u_{1}, v_{1})\}$ and $F \cap\{u_{1}, v_{1}, u_{2}, v_{2}\} = \{u_{1}, v_{2}\}$.

By the definition of $D'$, we have $\vert \partial_{D'}^{+}(F) \vert = \vert \partial_{D}^{+}(F) \vert $ if $\vert F \cap \{u_{1}, v_{1}, u_{2}, v_{2}\} \vert \neq2$. So, we may assume $\vert F\cap\{u_{1},v_{1},u_{2},v_{2}\} \vert =2$. In fact, also by the definition of $D'$, when $F\cap\{u_{1},v_{1},u_{2},v_{2}\} \in\{ \{u_{1},u_{2}\}, \{v_{1}, v_{2}\}\}$, $\vert \partial_{D'}^{+}(F) \vert = \vert \partial_{D}^{+}(F) \vert $ still holds. The other cases are illustrated as (ii)-(v). Thus Claim 1 must hold.

*Claim* 2. $\lambda(D')\geq\lambda(D)$.

By contradiction, we assume that $D'$ has an out-fragment *F* with $\vert \partial_{D'}^{+}(F) \vert < \lambda(D)$. By Claim 1 and since $\vert \partial_{D}^{+}(F) \vert \geq\lambda(D)$, we may assume that $\{u_{1},v_{1},u_{2},v_{2}\}\cap F=\{u_{1},v_{2}\}$. Thus $\vert \partial_{D}^{+}(F) \vert = \vert \partial_{D'}^{+}(F) \vert +1<\lambda(D)+1$. Since $\vert \partial_{D}^{+}(F) \vert \geq\lambda(D)$, we have $\vert \partial _{D}^{+}(F) \vert =\lambda(D)$, and so *F* is also an out-fragment of *D*. Since $u_{1}\in F_{1}\cap F$ and $u_{2}\notin F_{1}\cup F$, by a sub-modular inequality, we have

$$2\lambda(D)\leq \bigl\vert \partial_{D}^{+}(F_{1}\cap F) \bigr\vert + \bigl\vert \partial_{D}^{+}(F_{1}\cup F) \bigr\vert \leq \bigl\vert \partial_{D}^{+}(F_{1}) \bigr\vert + \bigl\vert \partial_{D}^{+}(F) \bigr\vert =2\lambda(D), $$

which implies $F_{1}\cap F$ is also an out-fragment of *D*, which contradicts the minimality of $F_{1}$. This completes the proof of Claim 2.

By choice (2)(a) of *D* and by Claim 2, $\lambda (D')=\lambda(D)$. Then, by Claim 1, $F_{1}$ is not an out-fragment in $D'$, and any out-fragment *F* of $D'$ is still an out-fragment of *D* unless either $\{u_{1},v_{1},u_{2},v_{2}\}\cap F=\{u_{1},v_{2}\}$ or $\{u_{1},v_{1},u_{2},v_{2}\}\cap F=\{u_{1},v_{2}\}$. If there is such an *F* such that *F* is an out-fragment in $D'$ but not in *D*, then without loss of generality we may assume $\{u_{1},v_{1},u_{2},v_{2}\}\cap F=\{u_{1},v_{2}\}$. Thus $\vert \partial_{D}^{+}(F) \vert = \vert \partial_{D'}^{+}(F) \vert +1=\lambda(D)+1$. Moreover, by the minimality of $F_{1}$ and $F_{2}$, we have $\vert \partial_{D}^{+}(F_{1}\cap F) \vert \geq\lambda(D)+1$. Thus, by a sub-modular inequality, we have

$$\begin{aligned} \lambda(D)&\leq \bigl\vert \partial_{D}^{+}(F\cup F_{1}) \bigr\vert \leq \bigl\vert \partial_{D}^{+}(F) \bigr\vert + \bigl\vert \partial_{D}^{+}(F_{1}) \bigr\vert - \bigl\vert \partial_{D}^{+}(F\cap F_{1}) \bigr\vert \\ &\leq\lambda(D)+ \lambda(D)+1- \bigl(\lambda(D)+1 \bigr)=\lambda(D). \end{aligned}$$

This implies $\vert \partial_{D}^{-}(\overline{F\cup F_{1}}) \vert = \vert \partial_{D}^{+}(F\cup F_{1}) \vert =\lambda(D)$. Then, by a sub-modular inequality again, we have

$$\begin{aligned} \bigl\vert \partial_{D}^{-}(\overline{F\cup F_{1}}\cap F_{2}) \bigr\vert &\leq \bigl\vert \partial_{D}^{-}(F_{2}) \bigr\vert + \bigl\vert \partial_{D}^{-}(\overline{F\cap F_{1}}) \bigr\vert - \bigl\vert \partial_{D}^{-}( \overline{F\cup F_{1}}\cup F_{2}) \bigr\vert \\ & \leq\lambda(D)+ \lambda (D)-\lambda(D)=\lambda(D), \end{aligned}$$

which implies $\vert \partial_{D}^{-}(\overline{F\cup F_{1}}\cap F_{2}) \vert =\lambda(D)$ contradicts to the minimality of $F_{2}$. Hence, every out-fragment of $D'$ is also an out-fragment of *D*. As $F_{1}$ is an out-fragment in *D* but not in $D'$, $\mathrm{fr}(D')<\mathrm{fr}(D)$, which contradicts choice (2)(b) of *D*. Therefore, *D* is *k*-arc-connected, and this completes the proof. □

By definition, the arc-connectivity of a digraph *D* cannot exceed $\min\{d^{+}_{D}(v), d^{-}_{D}(v): v \in V(D)\}$. A digraph *D* is *maximally arc-connected* if the arc-connectivity of *D* equals $\min\{d^{+}_{D}(v), d^{-}_{D}(v): v \in V(D)\}$. Applying Theorem 3.1 with $k=\min\{d_{1}^{+}, \ldots, d_{n}^{+}, d_{1}^{-}, \ldots, d_{n}^{-}\}$, we have the following corollary.

### Corollary 3.2

*Let* $\mathbf {d}=\{(d_{1}^{+},d_{1}^{-}),\ldots,(d_{n}^{+},d_{n}^{-})\}$ *be a multi*-*graphical sequence*. *Then* **d** *is also a degree sequence of some maximally arc*-*connected multi*-*digraph*.

## Degree sequence for multi-digraphs with prescribed connectivity

In this section, we consider the degree sequence of multi-digraphs with connectivity exactly *k*. Our method is to construct a new multi-digraph in $\langle\mathbf {d}\rangle$ from a *k*-arc-connected multi-digraph by reducing the arc-connectivity step by step. Moreover, by Corollary 3.2, we may assume that $k<\min_{1\leq i\leq n}\{d_{i}^{+}, d_{i}^{-}\}$.

### Theorem 4.1

*Let* $n\geq6, k\geq0$ *be two integers and* $\mathbf {d}=\{ (d_{1}^{+},d_{1}^{-}),\ldots,(d_{n}^{+},d_{n}^{-})\}$ *be a sequence of pairs of integers*. *Denote* $\delta_{1}=\min_{1\leq i\leq n} \{d_{i}^{+}, d_{i}^{-}\}$ *and* $\delta_{2}=\min_{1\leq i< j\leq n}$ $\{d_{i}^{+}+d_{j}^{+}, d_{i}^{-}+d_{j}^{-}\}$. *Then* **d** *is a degree sequence of some multi*-*digraph with connectivity exactly* *k* *if and only if each of the following hold*.

(i) $\delta_{1}\geq k$;
(ii) $\sum_{i=1}^{n} d_{i}^{+} =\sum_{i=1}^{n} d_{i}^{-}$;
(iii) *for* $j=1,\ldots, n$, $d_{j}^{+} +\alpha\leq\sum_{i\neq j} d_{i}^{-} +k$, *where* $\alpha=\delta_{2}$ *if* $k<\delta_{1}$ *and* $\alpha=\delta_{1}$ *if* $k=\delta_{1}$.

### Proof

First, we consider the necessity. Assume **d** is the degree sequence of some multi-digraph *D* with connectivity exactly *k*. By Theorem 1.2, (i) and (ii) hold. Suppose, to the contrary, that (iii) does not hold. Then there is a vertex $v_{j}$ of *D* such that $d^{+}(v_{j})=d_{j}^{+}\geq\sum_{i\neq j}d_{i}^{-}+k-\alpha+1$. It follows that $d^{+}(v_{j})+d^{-}(v_{j})\geq\sum_{i=1}^{n} d_{i}^{-} +k+1-\alpha = \vert A(D) \vert +k+1-\alpha$. This implies that there are at most $\alpha-k-1$ arcs not incident with $v_{j}$. On the other hand, as *D* has connectivity *k*, there exists $X\subseteq V(D)\setminus\{v_{j}\}$ such that either $d^{+}(X)=k$ or $d^{-}(X)=k$. Without loss of generality, we may assume the former. Then $d^{+}(X)\geq\sum_{v_{i}\in X} d^{+}(v_{i})-(\alpha-k-1)$. If $\vert X \vert \geq2$, then $\sum_{v_{i}\in X}d^{+}(v_{i})\geq\delta_{2}\geq\alpha $, and thus $d^{+}(X)\geq k+1$, a contradiction. So $\vert X \vert =1$ and thus $k=d^{+}(X)\geq \delta_{1}$, implying $\alpha=k=d^{+}(X)=\sum_{v\in X}d^{+}(v)$. Then again $d^{+}(X)\geq\sum_{v_{i}\in X}d^{+}(v_{i})-(\alpha-k-1)= k+1$, a contradiction. Hence (iii) holds.

Next, we consider the sufficiency. By Theorems 1.2 and 3.1, there is a *k*-arc-connected multi-digraph $D\in\langle\mathbf {d}\rangle$. If *D* has arc-connectivity *k*, then we are done. So we may assume that $\lambda(D)>k$, then we will construct a multi-digraph in $\langle\mathbf {d}\rangle$ with arc-connectivity exactly *k* from *D*. First, we need some claims.

Note that

$$\begin{aligned} \lambda(D) & =\min \bigl\{ d^{+}(X) |X\subseteq V(D), X, V(D)\setminus X\neq \emptyset \bigr\} \\ &=\min \bigl\{ d^{-}(X) |X\subseteq V(D), X, V(D)\setminus X\neq\emptyset \bigr\} . \end{aligned}$$

By a similarly analysis to Claim 1 in the proof of Theorem 3.1, it is easy to verify the following claim. In fact, in the tree operations in the following claim, $d^{+}(X)$ and $d^{-}(X)$ decrease at most 1 for any $\emptyset\neq X\subset V(D)$. The proof is easy and omitted here.

*Claim* 1. Each of the following holds.

(i) For any vertex disjoint two arcs $(u,v),(x,y)$, let $D\otimes\{(u,v),(x,y)\}$ have arc-connectivity at least $\lambda (D)-1$.
(ii) For any $x,y,z,w$ with $(x,y), (y,z), (z,w)\in A(D)$, $D-\{(x,y),(y,z), (z,w)\}+\{(x,z), (z,y), (y,w)\}$ has connectivity at least $\lambda(D) -1$.
(iii) For any $u,v,x,y,z$ with $(u,v), (x,y), (y,z)\in A(D)$, $D-\{(u,v),(x,y),(y,z)\}+\{(u,y), (y,v), (x,z)\}$ has arc-connectivity at least $\lambda(D)-1$.

Denote by

$$\lambda'(D)=\min \bigl\{ d^{+}(X)|X\subseteq V(D), \vert X \vert , \bigl\vert V(D)\setminus X \bigr\vert \geq 3 \bigr\} . $$

By the definition, $\lambda'(D)\geq\lambda(D)$ for any digraph *D*. Now, by Theorem 3.1, we may pick a multi-digraph $D\in \langle\mathbf {d}\rangle$ such that

3 $$ \textstyle\begin{array}{ll} \mbox{(a) $D$ is $k$-arc-connected.}\\ \mbox{(b) subject to (a), $\lambda'(D)$ is as small as possible.} \end{array} $$

Then we will construct a new digraph from *D* that meets our requirements.

By the choice of *D*, $\lambda(D) \geq k$. If $\lambda(D)=k$, then we are done and *D* is required. So we may assume that $\lambda(D)\geq k+1$. By the definition, let $X\subseteq V(D)$ so that $d^{+}(X)=\lambda'(D)$.

*Claim* 2. For any two arcs $(x_{1},y_{1})\in\partial^{+}(X), (y_{2},x_{2})\in\partial^{-}(X)$, either $x_{1}=x_{2}$ or $y_{1}=y_{2}$.

Suppose, to the contrary, that $x_{1}\neq x_{2}$ and $y_{1}\neq y_{2}$. Let $D'=D\otimes\{(x_{1},y_{1}), (y_{2},x_{2})\}$ and then by Claim 1, $D'\in \langle\mathbf {d}\rangle$ with arc-connectivity at least $\lambda (D)-1\geq k$ and $\lambda'(D')\leq d^{+}(X)-1=\lambda'(D)-1$, a contradiction to choice (3) of *D*.

*Claim* 3. For any two arcs $(x_{1},y_{1}), (x_{2},y_{2})\in\partial ^{+}(X)$ (or $\partial^{-}(X)$), either $x_{1}=x_{2}$ or $y_{1}=y_{2}$.

Suppose, to the contrary, that $x_{1}\neq x_{2}$ and $y_{1}\neq y_{2}$ and, without loss of generality, we may assume that $(x_{1},y_{1}), (x_{2},y_{2})\in \partial^{+}(X)$. As $\lambda'(D)\geq k+1\geq1$, $\partial^{-}(X)\neq \emptyset$. Let $(y_{3},x_{3})\in\partial^{-}(X)$. Then, by Claim 2, either $x_{3}=x_{2}$, $y_{3}=y_{1}$ or $x_{3}=x_{1}$, $y_{3}=y_{2}$. By symmetry, we may assume the former. Let $D'=D-\{(x_{1},y_{1}),(y_{1},x_{2}),(x_{2},y_{2})\}+\{(x_{1},x_{2}), (x_{2},y_{1}), (y_{1},y_{2})\}$. By Claim 1(ii), $D'\in\langle\mathbf {d}\rangle$ and has arc-connectivity at least $\lambda(D)-1\geq k$. However, $\lambda'(D')< \vert \partial^{+}(X) \vert =\lambda'(D)$, a contradiction to the choice of *D*. Claim 3 is proved.

By Claim 2 and Claim 3, it is easy to see that all arcs leaving from or interring to *X* are incident with a vertex, say *x*. We only consider the case $x\in X$, and the other case that $x\notin X$ can be dealt with similarly.

*Claim* 4. We may assume that $X\setminus\{x\}$ is an independent set of *D*.

Suppose, to the contrary, that there is an edge $(x_{1},x_{2})\in A(D[X\setminus\{x\}])$, then pick $y_{1},y_{2}\notin X$ such that $(y_{1},x), (x,y_{2})\in A(D)$. If $y_{1}\neq y_{2}$, then let $D'=D-\{(x_{1},x_{2}), (y_{1},x), (x,y_{2})\}+\{(x_{1},x), (x,x_{2}), (y_{1},y_{2})\}$ and thus $D'\in \langle\mathbf {d}\rangle$. By Claim 1(ii), $\lambda(D')\geq\lambda (D)-1\geq k$ and $\lambda(D')< \lambda'(D)$, a contradiction to choice (3) of *D*. So $y_{1}=y_{2}$. By the arbitrariness of $y_{1},y_{2}$, there is $y\notin X$ such that all arcs leaving from or interring to *X* are incident with *y*. Let $Y=V(D)\setminus X$ and $Y\setminus\{y\}$ is an independent set; otherwise, if there exists $(y_{1},y_{2})\in A(D[Y])$, then let $D''=D-\{(x_{1},x_{2}), (y_{1},y_{2}), (x,y), (y,x)\} +\{(x_{1},x), (x,x_{2}), (y_{1}, y), (y, y_{2})\}$, and it is easy to see that $D''$ has arc-connectivity at least $\lambda (D)-1\geq k$ and $\lambda'(D'')<\lambda'(D)$, a contradiction to choice (3) of *D*. So $Y\setminus\{y\}$ is an independent set. Thus, we may rename $Y, y$ as $X, x$ and Claim 4 follows.

As $\vert X \vert \geq3$, let $x_{1},x_{2}\in X\setminus\{x\}$. $m=\min\{ \vert A(D-X) \vert , d^{+}(x_{1})+d^{+}(x_{2}), d^{-}(x_{1})+d^{-}(x_{2})\}$. We will consider a sequence of digraphs $D_{0},D_{1},\ldots, D_{m}$, where $D_{0}=D$, and for $i=1,\ldots, m$, if $D_{i-1}$ is constructed, then let $D_{i}=D_{i-1}- \{(x_{1},x),(x,x_{2}), (u,v)\}+\{(u,x), (x,v), (x_{1},x_{2})\}$ or $D_{i}=D_{i-1}-\{(x_{2},x), (x,x_{1}), (u,v)\}+\{(u,x), (x,v), (x_{2},x_{1})\}$. By the choice of *m*, all $D_{i}$’s can be constructed although they may be not unique. It is easy to see that $D_{i}\in\langle\mathbf {d}\rangle$.

If $D_{m}$ has arc-connectivity at most *k*, then by Claim 3 there exists *i* such that $D_{i}$ has arc-connectivity exactly *k*, and we are done. So we may assume that $D_{m}$ is $(k+1)$-arc-connected. Then $m= \vert A(D-X) \vert <\delta_{x}$; otherwise, if $m=d^{+}(x_{1})+d^{+}(x_{2})$, then $\partial_{D_{m}}^{+}(\{x_{1},x_{2}\})=\emptyset$, a contradiction to the assumption that $D_{m}$ is $(k+1)$-arc-connected. A similar contradiction is obtained when $m=d^{-}(x_{1})+d^{-}(x_{2})$. Thus $m= \vert A(D-X) \vert $ and then $V(D)\setminus\{x,x_{1},x_{2}\}$ is an independent set in $D_{m}$.

If $k=\delta_{1}$, then $\alpha=\delta_{1}=k$ and by (iii), $d_{j}^{+}\leq\sum_{i\neq j}d_{i}^{-}$, and the result holds by Corollary 3.2. So we may assume that $k<\delta_{1}$ and thus $\alpha=\delta_{2}$. Let $u,v$ be two vertices so that $\delta_{2}=\min\{d^{+}(u)+d^{+}(v), d^{-}(u)+d^{-}(v)\}$. If $x\in\{u,v\}$, then $\delta_{2}< \min\{d^{+}(x), d^{-}(x)\}\leq\min\{d^{-}(x_{1})+d^{-}(x_{2}), d^{+}(x_{1})+d^{+}(x_{2})\}$, a contradiction. So $x\notin\{u,v\}$. Then continue to construct the sequence of digraphs $D_{0},\ldots, D_{m}, D_{m+1},\ldots, D_{2m}$ such that for $i=m+1,\ldots, 2m$, $D_{i}$ is obtained from $D_{i-1}$ by replacing an arc between $x_{1},x_{2}$ with a dipath of length 2 between $x_{1},x_{2}$ and replacing a dipath of length 2 between *u*, with an arc between $u,v$. Then, similarly to the above, we may assume that $D_{2m}\in\langle\mathbf {d}\rangle$ is $(k+1)$-arc-connected and $V(D)\setminus\{x,u,v\}$ is an independent set in $D_{2m}$.

Moreover, by (ii) and (iii), for any *j*, $d_{j}^{+} + d_{j}^{-}+\alpha\leq \sum_{i=1}^{n} d_{i}^{-}+k = \sum_{i=1}^{n} d_{i}^{+}+k$, and thus $d_{j}^{-}+\alpha\leq\sum_{i\neq j}d^{-}_{i} +k$. So, by symmetry, we may assume that $d^{+}(u)+d^{+}(v)\leq d^{-}(u)+d^{-}(v)$. Thus $\delta_{2}=d^{+}(u)+d^{+}(v)$. It follows that

$$\begin{aligned} \sum_{w\neq x}d^{-}(w) - d^{+}(x) = {}& \bigl\vert A \bigl(D_{2m} \bigl[\{u,v\} \bigr] \bigr) \bigr\vert \\ = {}&d^{+}(u) + d^{+}(v) - \bigl\vert \partial_{D_{2m}} \bigl(\{u,v\} \bigr) \bigr\vert \\ \leq{}&\alpha-k-1. \end{aligned}$$

This implies that there is *j* such that $d_{j}^{+}+\alpha\geq\sum_{i\neq j}d_{i}^{-}+k+1$, a contradiction to (iii). The proof is completed. □

If Theorem 4.1(i), (ii) holds, then by Theorem 1.2 $\langle\mathbf {d}\rangle\neq\emptyset$. Furthermore, if Theorem 4.1(iii) does not hold, then there are no digraphs in $\langle\cdot\rangle$ that have arc-connectivity exactly *k*. In other words, all digraphs in $\langle\mathbf {d}\rangle$ are $(k+1)$-arc-connected.

### Corollary 4.2

*Let* $\mathbf {d}=\{(d_{1}^{+}, d_{1}^{-}),\ldots, (d_{n}^{+}, d_{n}^{-})\}$ *be a sequence of integer pairs*. *Denote* $\delta_{1}=\min_{1\leq i\leq n} \{ d_{i}^{+}, d_{i}^{-}\}$ *and* $\delta_{2}=\min_{1\leq i< j\leq n}$ $\{d_{i}^{+}+d_{j}^{+}, d_{i}^{-}+d_{j}^{-}\}$. *If each of the following holds*, *then any digraphs in* $\langle\mathbf {d}\rangle$ *are* *k*-*arc*-*connected*.

(i) $\delta_{1}\geq k$;
(ii) $\sum_{i=1}^{n} d_{i}^{+} =\sum_{i=1}^{n} d_{i}^{-}$;
(iii) *there exists some* *j* *such that* $d_{j}^{+} +\alpha\geq\sum_{i\neq j} d_{i}^{-} +k$, *where* $\alpha=\delta_{2}$ *if* $k<\delta_{1}$ *and* $\alpha=\delta_{1}$ *if* $k=\delta_{1}$.

## Conclusions

In this paper, sufficient and necessary conditions for a sequences of pairs of integers have been studied. For a sequence $\mathbf {d}=\{ (d_{1}^{+},d_{1}^{-}), \ldots, (d_{n}^{+}, d_{n}^{-})\}$ of pairs of integers, we give a sufficient and necessary condition of **d** to have a *k*-arc-connected realization by using some inequalities of these integers. As a consequence, we deduce a sufficient and necessary condition of **d** to have a max-arc-connected realization. Also, when $n\geq6$, we give a sufficient and necessary condition of **d** to have a realization *D* that has arc-connectivity exactly *k*. These results extend a similar result from undirect graphs into directed graphs.
